# Supplementary material for: Paternal imprinting of dosage-effect defective1 contributes to seed weight xenia in maize
Source: Nat Commun. 2022 Sep 13;13:5366. doi: 10.1038/s41467-022-33055-9 (PMC9470594; doi:10.1038/s41467-022-33055-9)
Supplement: Supplementary file 1 — Supplementary Information [file 41467_2022_33055_MOESM1_ESM.pdf]

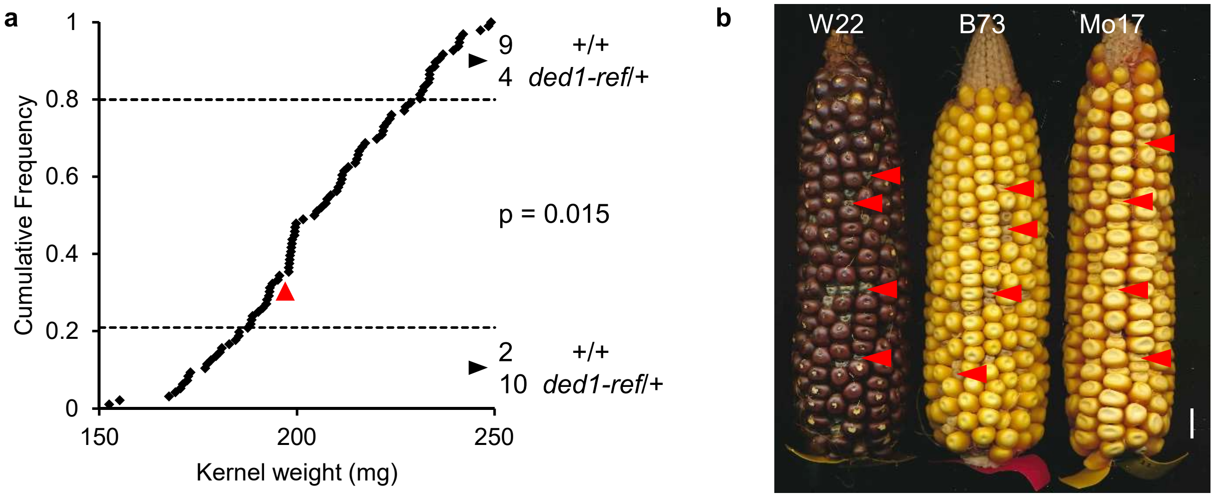

c

genetic background	W22			B73 (BC_7S_1)		
ear	1	2	3	1	2	3
Normal	198	227	243	264	260	252
mutant	76	79	74	80	77	89
χ2	1.09	0.11	0.46	0.56	0.83	0.22
p-value	0.30	0.74	0.50	0.46	0.36	0.64

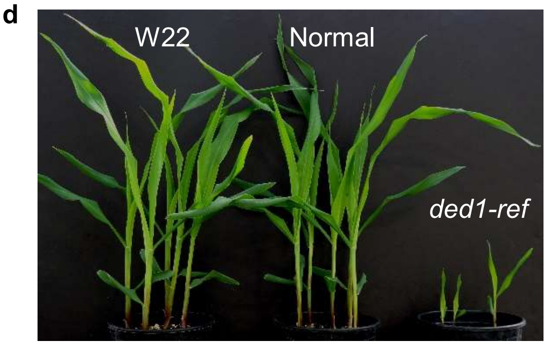

Supplementary Figure 1. **Identification of the *ded1-ref* mutant. a** Cumulative distribution function of individual kernel weight for normal kernels from the *ded1-ref*/+ M_3 ear. Red arrowhead indicates a 2.3 mg kernel weight increase. Arrowheads indicate the number of normal and segregating self-pollinations recovered from the heaviest 20 and lightest 20 kernels that were planted in separate cultures. The 0.015 p-value is for a two-tailed Fisher's exact test without correction for multiple testing. **b** Self-pollinated ears of *ded1-ref*/+ plants in W22, B73 (BC_4S_1), and Mo17 (BC_4S_1) genetic backgrounds. Scale bar is 1 cm. **c** Segregation of *ded1-ref* mutant kernels in three independent self-pollinations in the W22 and B73 genetic backgrounds. χ^2 statistics and p-values are for a 3:1 segregation ratio without correction for multiple tests. **d** Seedling phenotype of W22, normal siblings, and *ded1-ref* homozygous mutants.

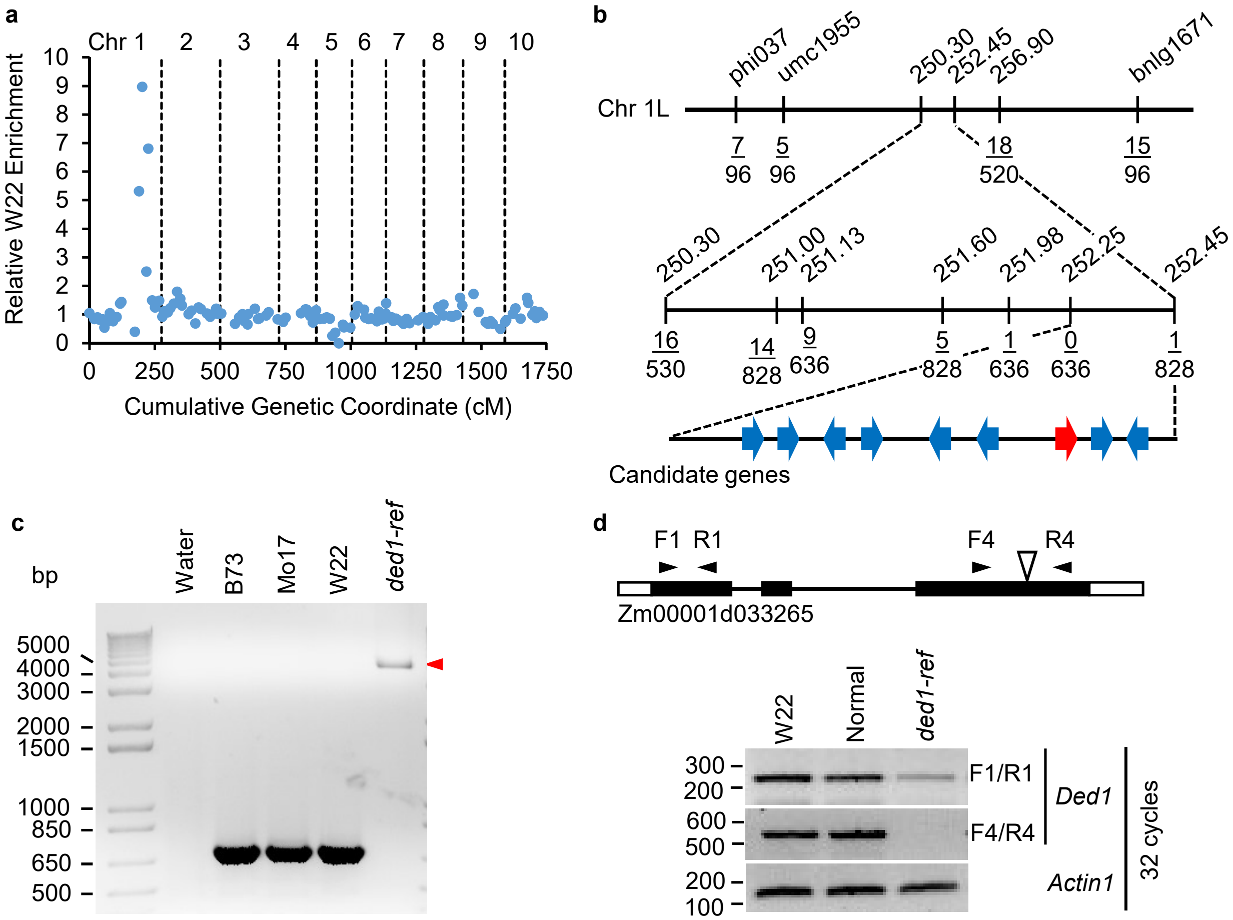

Supplementary Figure 2. **Cloning of the *ded1* locus. a** Bulked segregant analysis mapping of a 100 kernel pool of *ded1-ref* mutants from a B73 x W22 F_2 mapping population. Scatterplot shows enrichment for the W22 allele for each SNP marker relative to a 1:1 mix of B73:W22 DNA. **b** Fine mapping of the *ded1* locus. Molecular markers are indicated above physical maps and observed recombinants/genotyped progeny for each marker are below physical maps. Primers for numbered markers follow the convention of ufIDP1_250.30 in Table S5. Gene models in the fine-map interval are denoted with blue arrows with the *ded1* locus in red. **c** Amplification of the *ded1* locus from B73, Mo17, W22, and homozygous *ded1-ref* genomic DNA. The red arrowhead indicates the larger product due to the LTR retrotransposon insertion in *ded1-ref*. The complete LTR insertion was amplified three times. PCR with these specific genomic DNA samples and primers was completed once. **d** RT-PCR analysis of *Ded1* exon 1, *Ded1* exon 3, and *Actin1* in 12 DAP W22, normal sibling, and *ded1-ref* endosperm tissue. Gene model schematic shows the positions of the Ded1-F1/Ded1-R1 and Ded1-F4/Ded1-R4 primer sets with black arrowheads. The *ded1-ref* retrotransposon insertion is indicated with a white arrowhead. The RT-PCR was repeated three times with similar results.

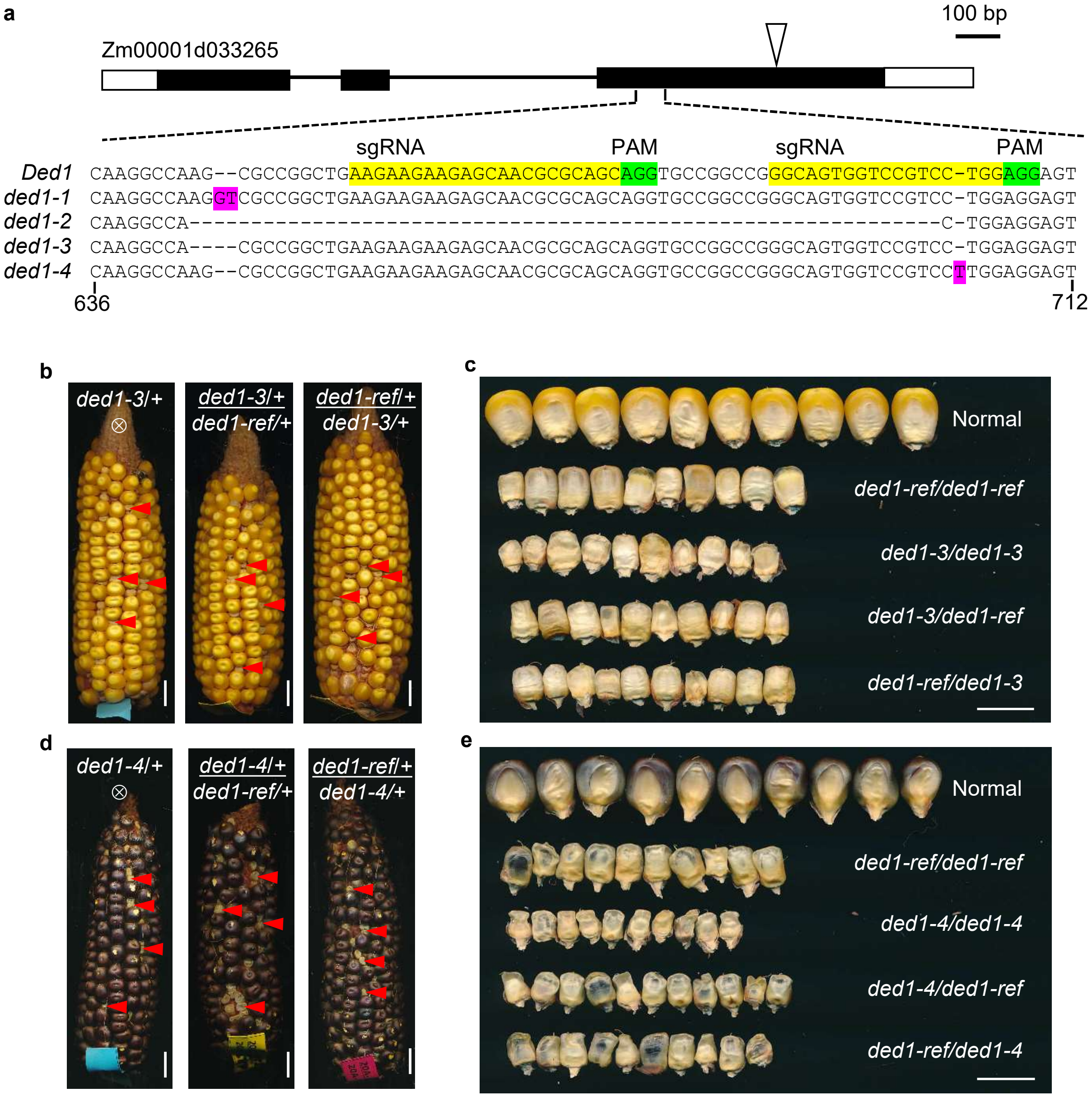

Supplementary Figure 3. **Cas9-induced lines and complementation test. a** Gene model schematic and multiple sequence alignment of the Cas9-induced mutant alleles of *ded1*. The sgRNA and PAM sites are highlighted in yellow and green, respectively. Base insertions are highlights in fuchsia. **b** Self-pollination of *ded1-3*/+ and reciprocal crosses with *ded1-ref*/+ in the B73 genetic background. Red arrowheads indicate mutant kernels. **c** Mature kernel phenotypes of normal sibling, *ded1-ref* mutants, *ded1-3* mutants, and reciprocal heteroallelic crosses in a B73 genetic background. Germinal face of 10 kernels is shown for each genotype. **d** Self-pollination of *ded1-4*/+ and reciprocal crosses with *ded1-ref*/+ in the W22 genetic background. Red arrowheads indicate mutant kernels. **e** Mature kernel phenotypes of normal sibling, *ded1-ref* mutants, *ded1-3* mutants, and reciprocal heteroallelic crosses in a W22 genetic background. Germinal face of 10 kernels is shown for each genotype. Scale bars are 1 cm.

a

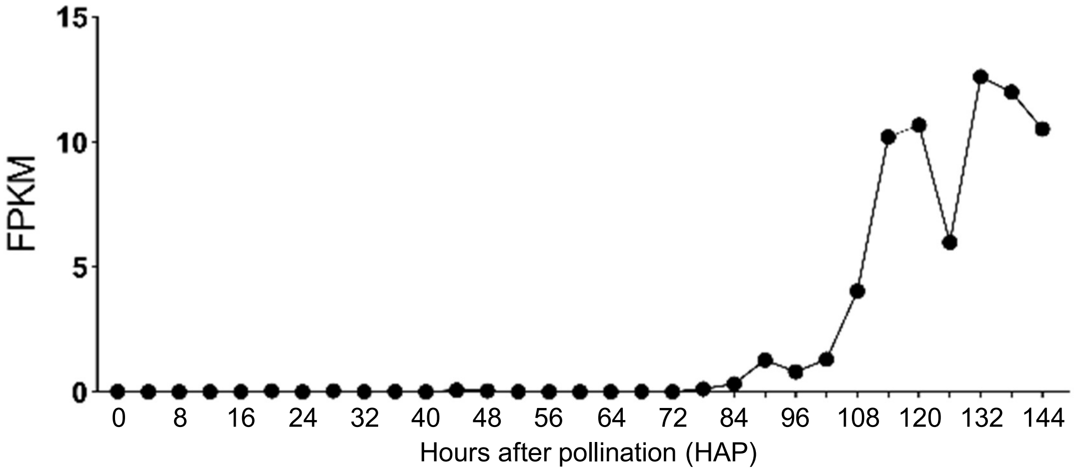

b

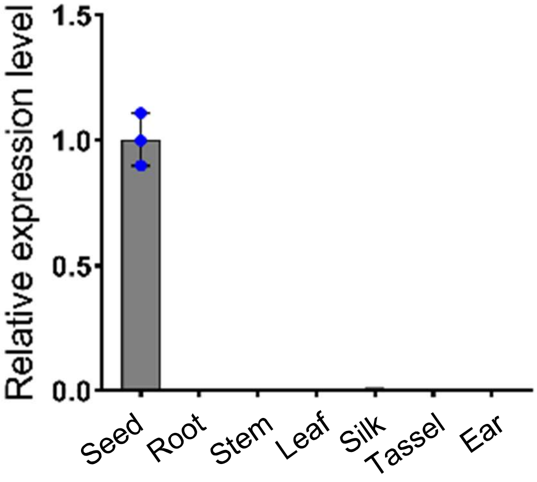

Supplementary Figure 4. **Expression pattern of *Ded1*. a** Expression pattern of *Ded1* in developing seed from 0-144 hours after pollination (HAP). FPKM values were based on public maize transcriptome data[1]. **b** qRT-PCR analysis of *Ded1* in W22 tissues. The qPCR product is the same exon 1 product as in Fig. 1h and 2a. Transcript levels were normalized to maize 18s rRNA.

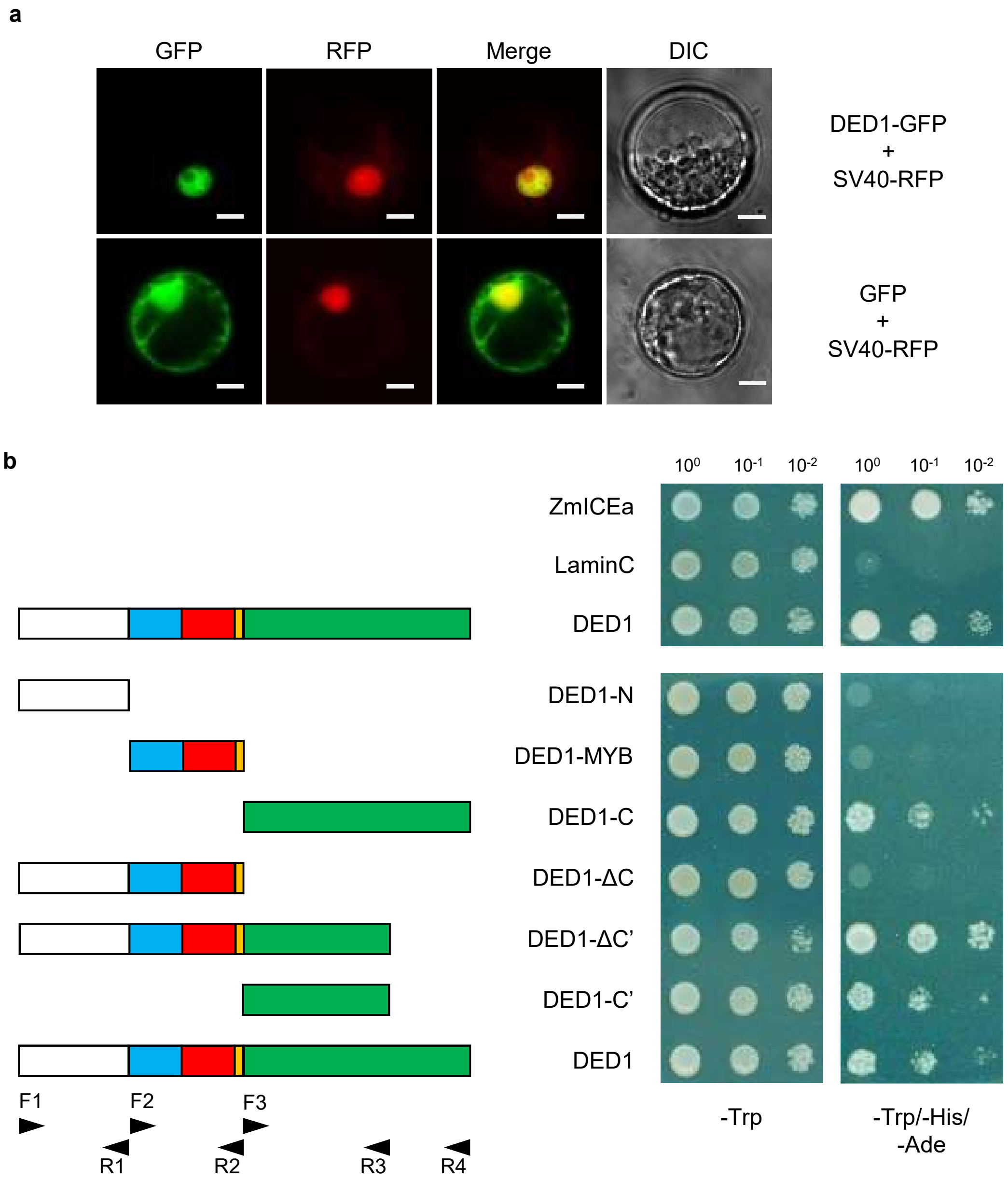

Supplementary Figure 5. ***Ded1* encodes a transcription factor. a** Transient co-expression of DED1-GFP and the nuclear marker, SV40-RFP, in maize protoplasts. Co-expression of GFP and SV40-RFP shows cytosolic and nuclear subcellular domains. Scale bars are 5 mm. Data are representative of five independently co-transformed cells imaged. **b** Yeast autoactivation assays using GAL4 DNA-BD constructs and query proteins were expressed as C-terminal fusions in AH109 cells and selected on SD/-Trp and SD/-Trp/-His/-Ade. ZmICEa is a known transcriptional activator[2]. LaminC is a non-activating control. Full-length DED1 and truncations are diagrammed in the schematic with the R2 (blue) and R3 (red) MYB DNA binding domains, the nuclear localization signal (yellow), and C-terminal acidic domain (green). Arrowheads indicate primers used to clone the truncations from the *Ded1* cDNA. Primers are in Table S5 named with the convention DED1-BD-F1.

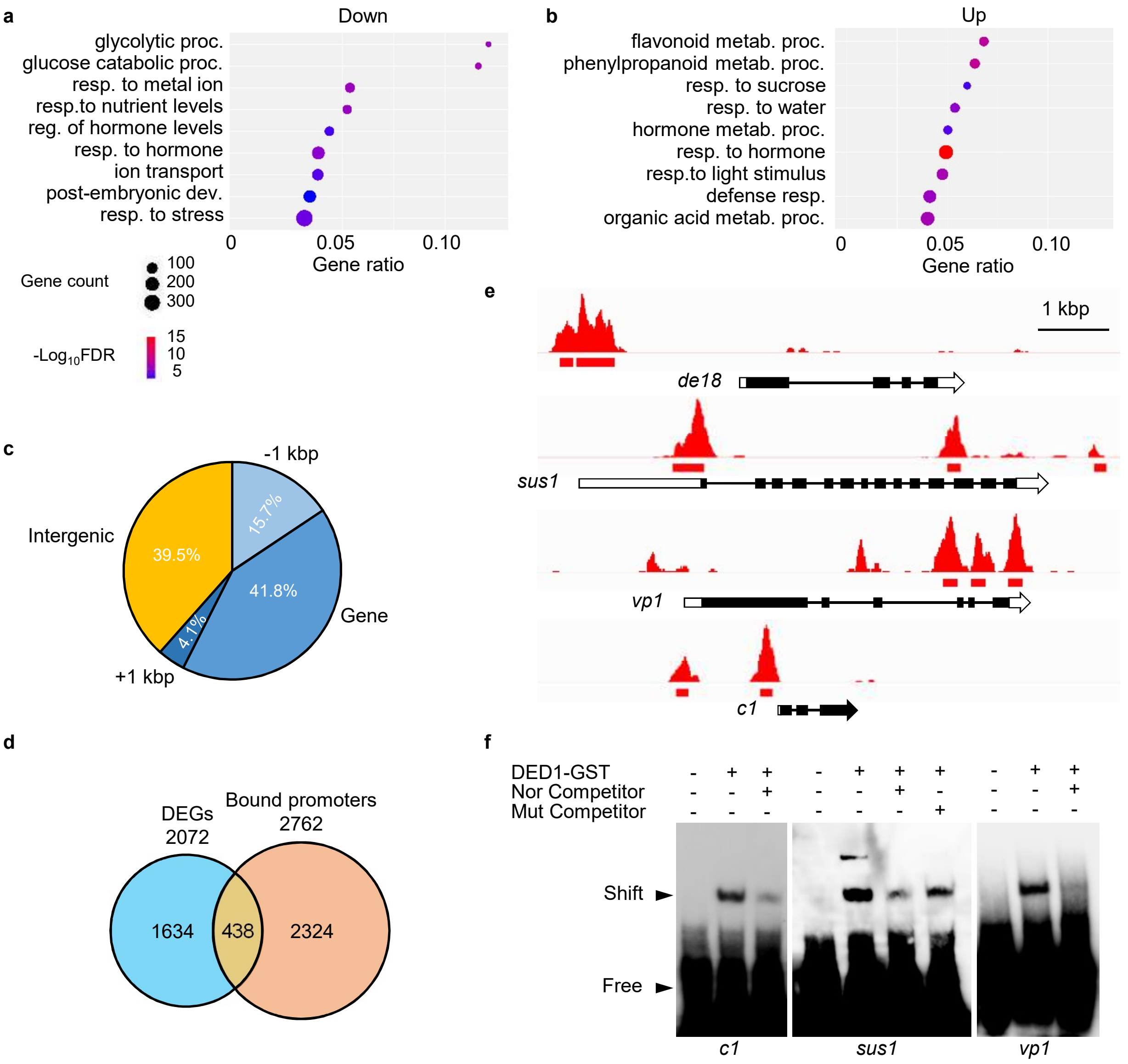

Supplementary Figure 6. **Identification of DED1 direct target genes. a** Enriched GO-terms in DEGs that show reduced expression in *ded1* mutants. **b** Enriched GO-terms in DEGs that show increased expression in *ded1* mutants. For **a** and **b**, symbol color is a heat map scale for statistical significance and symbol size is proportional to the number of DEGs for the GO term. The x axis indicates the enrichment ratio for DEGs relative to all genes with the GO term. **c** Distribution of DED1 DAP-seq peaks in the maize genome based on localization of peak summits. Genomic locations were defined as -1 kbp to the TSS; gene sequences from the TSS to TTS, 1 kbp downstream of the TTS; and intergenic regions. **d** Venn diagram of DEGs ($q<0.05$, FC>2, and TPM>1) and genes bound by HALO-DED1 from -1 to +0.1 kbp of the TSS. Intersecting genes were classified as direct targets of DED1. **e** DAP-seq read density of selected genes. Arrows indicate the TSS and direction of transcription for the gene. **f** EMSA with DED1-GST purified protein and labelled *c1*, *sus1*, and *vp1* promoter fragments. Normal (Nor) or mutant (Mut) competitor probes were added at 100-fold greater concentration than the labelled probe. EMSA for *sus1* was completed twice. EMSA for *c1* and *vp1* was completed once.

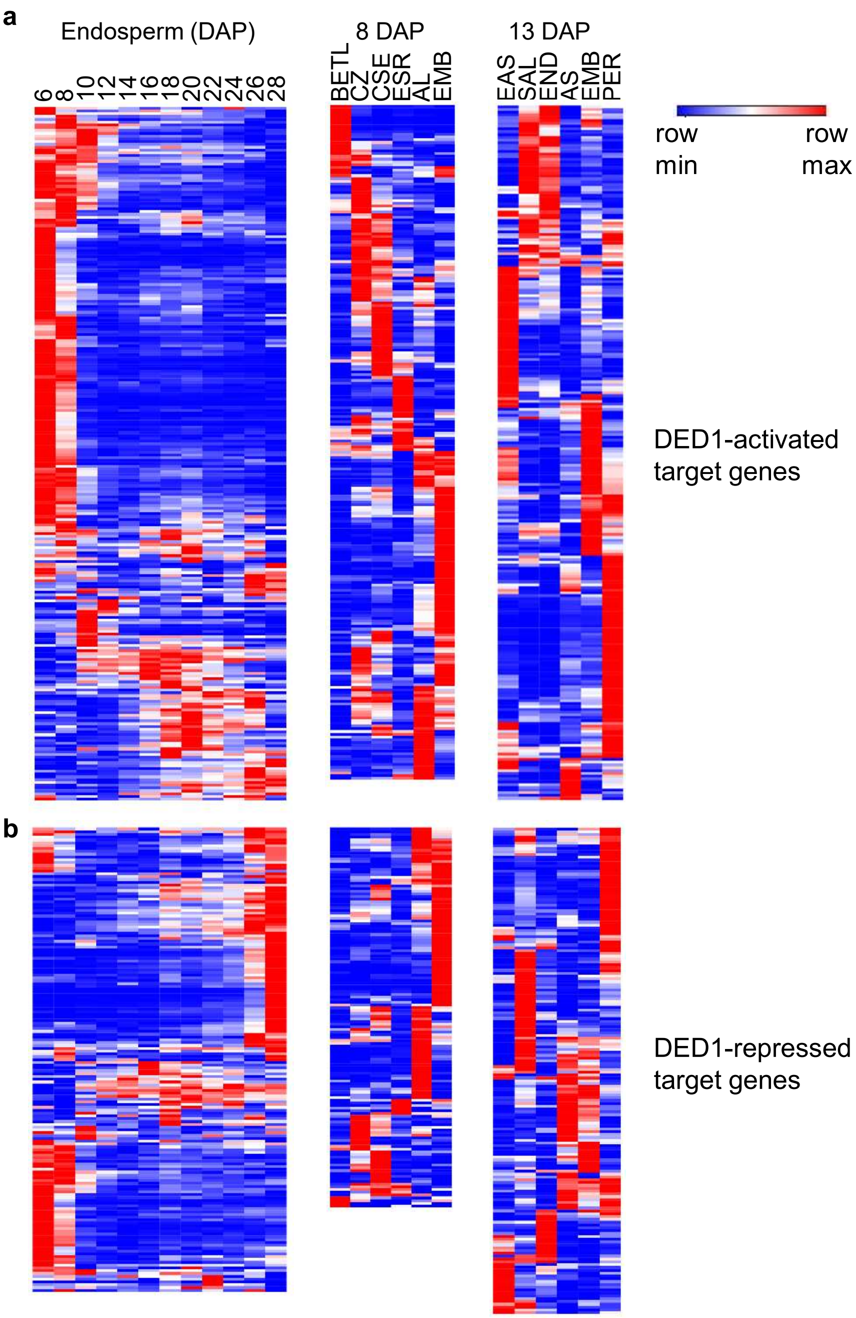

Supplementary Figure 7. **Kernel expression patterns of DED1 direct target genes. a** and **b**, Heat maps showing relative expression levels of DED1-activated (**a**) and DED1-repressed (**b**) target genes from published transcriptome data[3,4,5]. Data from each study was hierarchically clustered using Euclidean distance independently, and the heat map scale is independent for each gene to illustrate the temporal or tissue expression maximum for a gene within a study. Endosperm compartment abbreviations are: BETL, basal endosperm transfer layer; CZ, conducting zone; CSE, central starchy endosperm; ESR, embryo-surrounding region; AL, aleurone layer; EMB, embryo; EAS, endosperm adjacent to scutellum; SAL, scutellar aleurone layer; END, endosperm; AS, apical scutellum; and PER, Pericarp.

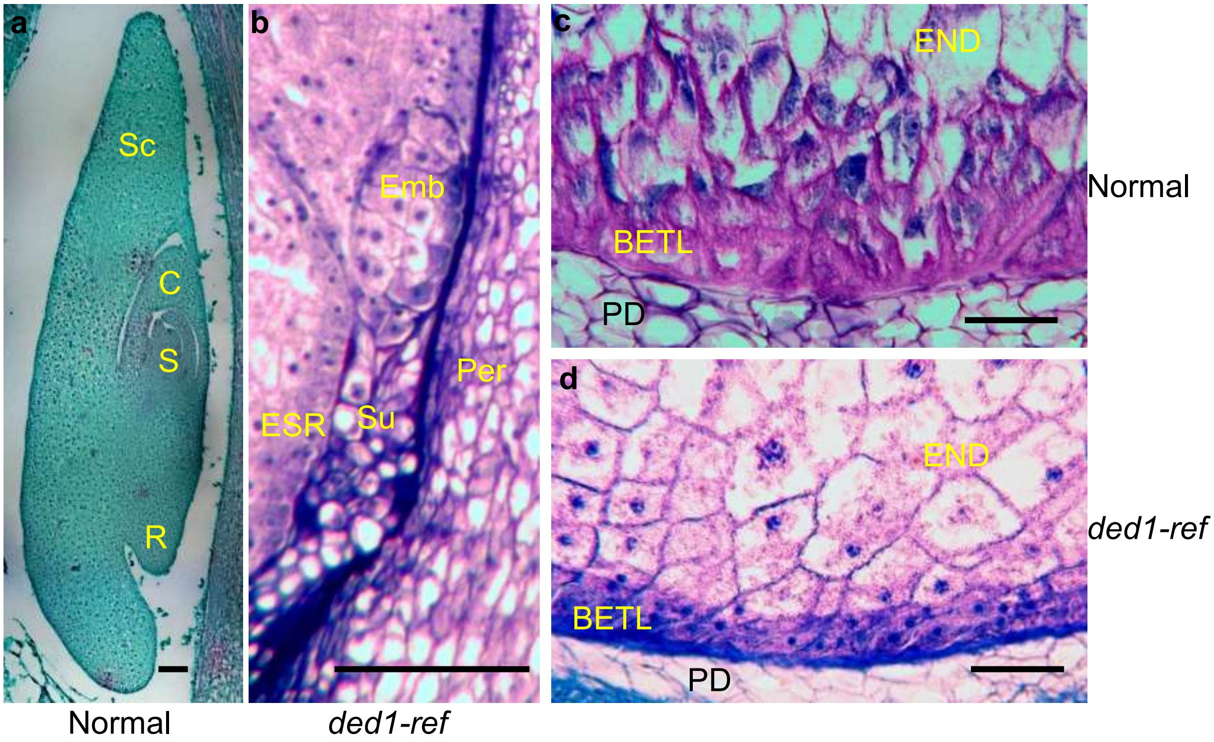

Supplementary Figure 8. **Developmental defects in 12 DAP *ded1-ref* kernels. a** and **b**, Transverse sections of WT (**a**) and *ded1-ref* (**b**) embryo. Abbreviations for morphological structures are: Sc, scutellum; C, coleoptile; S, shoot apex; R, root; Emb, embryo; Su, suspensor; ESR, embryo surrounding region, and Per, pericarp. Scale bars are 200 µm. **c** and **d,** WT (**c**) and *ded1-ref* (**d**) BETL region with endosperm (END) and maternal pedicel (PD). Scale bars are 50 µm. Images are representative of 24 kernels sectioned from three self-pollinated ears (8 kernels per ear).

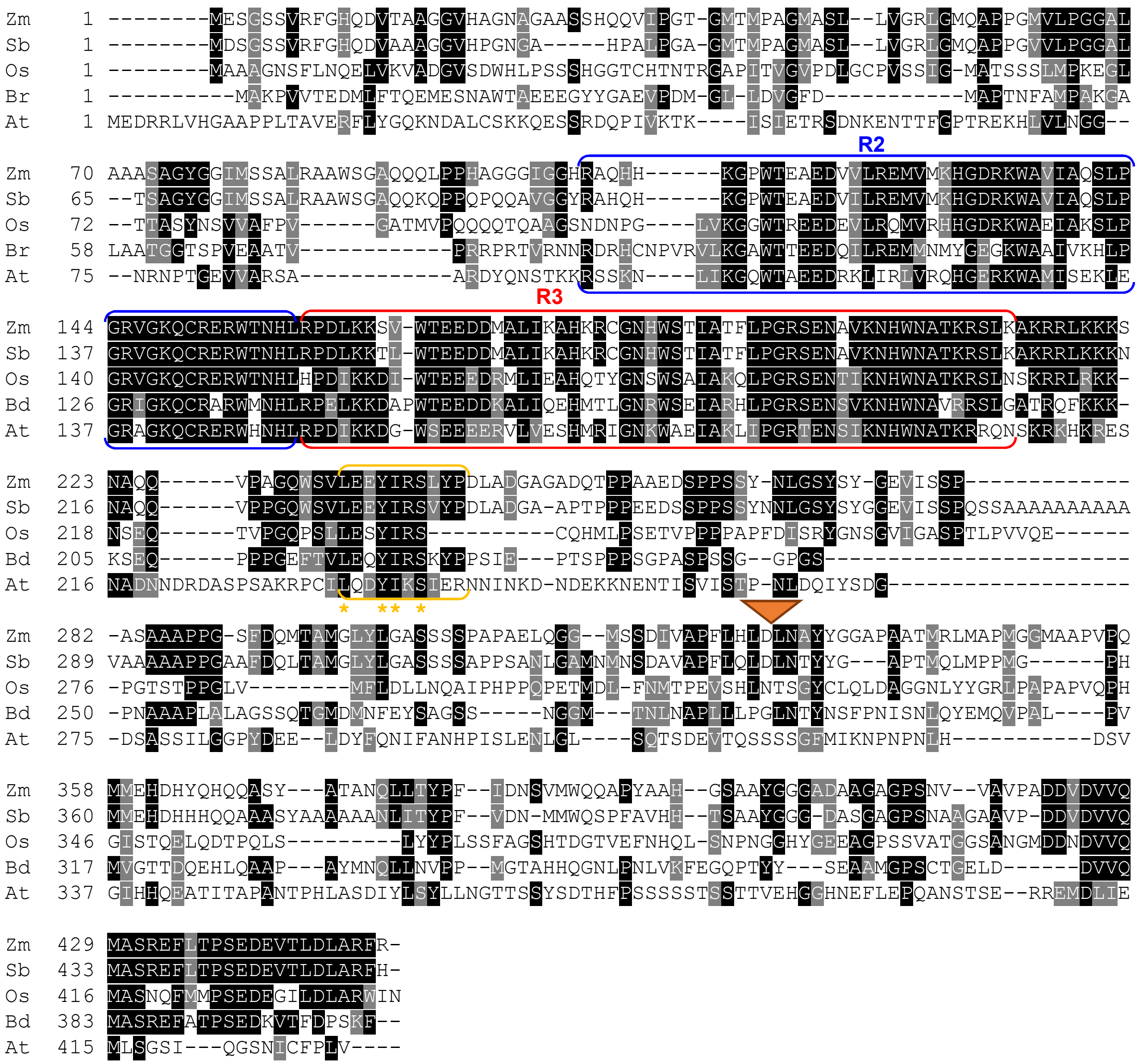

Supplementary Figure 9. **Protein sequence alignment of DED1 orthologs.** DED1 orthologs from *Zea mays* (Zm, NP_001132209.1), *Sorghum bicolor* (Sb, XP_002464189.1), *Oryza sativa* (Os, XP_015645903.1), *Brachypodium distachyon* (Bd, XP_024314632.1), and *Arabidopsis thaliana* (At, NP_568891.1) were aligned with ClustalW using default settings. Blue and red brackets are the R2 and R3 MYB DNA binding domains, respectively. Yellow asterisks are completely conserved sites within the subgroup 25 domain denoted by yellow brackets[6]. The triangle indicates the *ded1-ref* transposon insertion site.

Supplementary Table 1

Primer Name	Primer Sequence (5'→3')
**SSR and InDel primers for genetic map in Supplementary Fig. 2b**	
phi037-L	CCCAGCTCCTGTTGTCGGCTCAGAC
phi037-R	TCCAGATCCGCCGCACCTCACGTCA
umc1955-L	GCCAAGGTGGGTCTGGCTAT
umc1955-R	ACCACCTTGTCCGTATCCTTCAC
bnlg1671-L	TCACGATCAGCAAGCAATTC
bnlg1671-R	CCCCACCAACCTTAGAGTCA
ufIDP1_250.30-L	TTGTAGGGGTTTCTGCCTTC
ufIDP1_250.30-R	CATGGCACCACGACAGTAAA
ufIDP1_251.09-L	AGGGAATTTCTGCTGGACCT
ufIDP1_251.09-R	GGCAGTTGCATCACTGAAAG
ufIDP1_251.13-L	GCATGTGCATGAATGAGGGA
ufIDP1_251.13-R	GCATGCATCGTGACAAAAGT
ufIDP1_251.60-L	CCATAGCAAAATCTGGACGTT
ufIDP1_251.60-R	CCTCCCCCTCTTCATATCAT
ufIDP1_251.98-L	ACTAGTGATGGGTGGGTGGA
ufIDP1_251.98-R	GGGAATATTTTGGTTGCCCTA
ufIDP1_252.25-L	CATCAGCAAAGTTCGACAGC
ufIDP1_252.25-R	TCGAATGATGAAGAAACTTCCA
ufIDP1_252.45-L	ACGACCATTCTCCATTCTCG
ufIDP1_252.45-R	CAATGCGCACGTATAACTCG
***ded1* locus primers for Fig. 1, 2, and Supplementary Fig. 2c**	
MYB73-F15	AATGGAGTCCGGGAGCTCG
MYB73-RT-R4	TAGCTGCTGTGTCAACGGAACCT
Ded-30-F5	AGAGATGGACCAACCACCTG
MYB73-F6-A	CTGGAGGAGTACATCCGCA
MYB73-CAPS-L1	GACCTCAACGCCTACTACGG
MYB73-CAPS-R1	GCTGTGTCAACGGAACCTG
MYB73-R5	TTGCAGATAGCGTGCGTACT
MYB73-F6-A	CTGGAGGAGTACATCCGCA
LTR-F2	TAATAGATGTGCGCGCTGTT
MYB73-R9	GTCCAGGTGCAGGAACGG
**RT-PCR primers for Supplementary Fig. 2d**	
Ded1-F1	GGCGCTGCTTCTTCTCATCA
Ded1-R1	CAGCTGCTGCTGAGCCC
Ded1-F4	GAGTACATCCGCAGCCTGT
Ded1-R4	AACTCCCTGGAGGCCATCTG
ZmActin1-F	ATGGTCAAGGCCGGTTTCG
ZmActin1-R	TCAGGATGCCTCTCTTGGCC

qRT-PCR primers used in Fig. 1, 2, 4, and Supplementary Fig. 4b

Ded1-Fig1h-F4	GGAGCTCGGTGCGTTTCG
Ded1-Fig1h-R4	CGGGATCACCTGCTGATGAGA
18srRNA-qPCR-F	CCATCCCTCCGTAGTTAGCTTCT
18srRNA-qPCR-R	CCTGTCGGCCAAGGCTATATAC
Ded1-Fig4a-F1	GCAGCTTTGACCAGATGACC
Ded1-Fig4a-R1	GTAGTAGGCGTTGAGGTCCAG
fl3-qRT-F1	TGCACCCAGTGTGAGACCAA
fl3-qRT-R1	CGGACACCCTCACAACGTCA
de18-qRT-F1	GTGGCTTAAGGACGGTGGGA
de18-qRT-R1	CACGAGAGATGCCAGCCAGT
tcrr1-qRT-F1	AGGTGCCATGGAAGCATTGG
tcrr1-qRT-R1	TGGCGATTCCTTCACTTCCCT
az22z5-qRT-F1	ATACCGGCATGTGCAACTTC
az22z5-qRT-R1	TTGTATTGCCTATGTTTTGACCA
a2-qRT-F1	GCTACACCAGCGTCCTCCAC
a2-qRT-R1	TCGCAGAAGACGACCCAGGA
sweet4c-qRT-F1	GCCCTTGTGCTCTTCCTCTC
sweet4c-qRT-R1	CCGCACGCTTCTTCCAGATG

Primers for cloning constructs in Supplementary Fig. 5

GFP-6	CGACTCTAGAGGATCCATGGTGAGCAAGGGCGAG
GFP-7	ATTCGAGCTGGTCACCTTACTTGTACAGCTCGTCCATGC
73ORF-F4	CGACTCTAGAGGATCCATGGAGTCCGGGAGCTCG
73ORF-R4	TGCTCACCATGGATCCACGGAACCTGGCCAGGTCCA
RFP-F1	atgCcgaagaagaagaggaaggttATGGCCTCCTCCGAGGACGT
RFP-R2	ATTCGAGCTGGTCACCTTAGGCGCCGGTGGAGTG
RFP-F2	CGACTCTAGAGGATCCATGCCGAAGAAGAAGAGG
ICE1-BD-F	GAGGCCGAATTCATGGACGACTCGGCGGAG
ICE1-BD-R	CTGCAGGTCGACCATTGCGTTGTGGAGGCC
DED1-BD-F1	GAGGCCGAATTCATGGAGTCCGGGAGC
DED1-BD-R1	CTGCAGGTCGACCGCCCTATGCCCGCC
DED1-BD-F2	GAGGCCGAATTCATGCAGCACCACAAGGGC
DED1-BD-R2	CTGCAGGTCGACCTTCTTCAGCCGGCG
DED1-BD-F3	GAGGCCGAATTCATGAAGAGCAACGCGCAG
DED1-BD-R3	CTGCAGGTCGACGTCCAGGTGCAGGAA
DED1-BD-R4	CTGCAGGTCGACACGGAACCTGGCCA
pGEX-4T-1-EcoRI	TCCCCGGAATTCATGGAGTCCGGGAGC
pGEX-4T-1-XhoI	CGGCCGCTCGAGACGGAACCTGGCCA

EMSA oligos for Fig. 3e and Supplementary Fig. 6f

Vp1-F	GTCCTTGAAACTGCAATCCAAATCTACGCAACCGTTTTGCATGCCACTGTTTGTA
Vp1-R	TACAAACAGTGGCATGCAAAACGGTTGCGTAGATTTGGATTGCAGTTTCAAGGAC
C1-F	CATCATTCGATCAGTTTTCGTTCTGATGCAGTTTTCGATAAATGC
C1-R	GCATTTATCGAAAACTGCATCAGAACGAAAACTGATCGAATGATG
SUS1-F	CTGAACCGTTTTTCGGTTTCAGTTTTCTCTGCTGACTGAAGCTTC
SUS1-R	GAAGCTTCAGTCAGCAGAGAAAACTGAAACCGAAAAACGGTTCAG
SUS1-mut-F	CTGAACCGTTTTTCGGTTTAGACCTTCTCTGCTGACTGAAGCTTC
SUS1-mut-R	GAAGCTTCAGTCAGCAGAGAAGGTCTAAACCGAAAAACGGTTCAG
FL3-F	TGTACAAATATTTCTACTGACAGTTTTTAAGAAACCACCTGTGGA
FL3-R	TCCACAGGTGGTTTCTTAAAAACTGTCAGTAGAAATATTTGTACA
FL3-mut-F	TGTACAAATATTTCTACTGAAGACCTTTAAGAAACCACCTGTGGA
FL3-mut-R	TCCACAGGTGGTTTCTTAAAGGTCTTCAGTAGAAATATTTGTACA

Supplementary References

1. F. Yi *et al.*, High Temporal-Resolution Transcriptome Landscape of Early Maize Seed Development. *Plant Cell* **31**, 974-992 (2019).
2. A. Grimault *et al.*, ZmZHOUPI, an endosperm-specific basic helix-loop-helix transcription factor involved in maize seed development. *Plant J.* **84**, 574-586 (2015).
3. N. M. Doll *et al.*, Transcriptomics at Maize Embryo/Endosperm Interfaces Identifies a Transcriptionally Distinct Endosperm Subdomain Adjacent to the Embryo Scutellum. *Plant Cell* **32**, 833-852 (2020).
4. J. Zhan *et al.*, RNA sequencing of laser-capture microdissected compartments of the maize kernel identifies regulatory modules associated with endosperm cell differentiation. *Plant Cell* **27**, 513-531 (2015).
5. J. Chen *et al.*, Dynamic transcriptome landscape of maize embryo and endosperm development. *Plant Physiol.* **166**, 252-264 (2014).
6. R. Stracke, M. Werber, B. Weisshaar, The R2R3-MYB gene family in Arabidopsis thaliana. *Curr. Opin. Plant Biol.* **4**, 447-456 (2001).
