# Supplementary material for: Paternal imprinting of dosage-effect defective1 contributes to seed weight xenia in maize
Source: Nat Commun. 2022 Sep 13;13:5366. doi: 10.1038/s41467-022-33055-9 (PMC9470594; doi:10.1038/s41467-022-33055-9)
Supplement: Supplementary file 3 — Description of Additional Supplementary Files [file 41467_2022_33055_MOESM3_ESM.pdf]

Description of Additional Supplementary Files

File Name: Supplementary Data 1
Description: DEGs in ded1-ref endosperm. DESeq2 statistics are reported for 2,072 DEGs identified from 12 DAP RNA-seq comparing pooled normal versus ded1-ref mutant endosperm tissues.

File Name: Supplementary Data 2
Description: DED1 potential target genes with DAP-seq peaks in the promoter region. A total of 7,069 DAP-seq peaks were identified within -1 kbp and +100 bp of annotated transcriptional start sites in the maize B73_v3 genome. The data set represents 5,860 gene models of which 2,762 genes were tested for differential expression in the RNA-seq experiment.

File Name: Supplementary Data 3
Description: Analysis of direct DED1 target genes. 438 DEGs have DED1 binding sites in promoter regions. The table integrates abbreviated results from RNA seq, DAP-seq, public transcriptome analysis, and gene annotations.
