# Supplementary material for: Paternal imprinting of dosage-effect defective1 contributes to seed weight xenia in maize
Source: Nat Commun. 2022 Sep 13;13:5366. doi: 10.1038/s41467-022-33055-9 (PMC9470594; doi:10.1038/s41467-022-33055-9)
Supplement: Supplementary file 7 — Reporting Summary [file 41467_2022_33055_MOESM7_ESM.pdf]

nature portfolio

Corresponding author(s): A. Mark Settles

Last updated by author(s): Aug 28, 2022

Reporting Summary

Nature Portfolio wishes to improve the reproducibility of the work that we publish. This form provides structure for consistency and transparency in reporting. For further information on Nature Portfolio policies, see our Editorial Policies and the Editorial Policy Checklist.

Statistics

For all statistical analyses, confirm that the following items are present in the figure legend, table legend, main text, or Methods section.

n/a	Confirmed	
☐	☒	The exact sample size (*n*) for each experimental group/condition, given as a discrete number and unit of measurement
☐	☒	A statement on whether measurements were taken from distinct samples or whether the same sample was measured repeatedly
☐	☒	The statistical test(s) used AND whether they are one- or two-sided<br>*Only common tests should be described solely by name; describe more complex techniques in the Methods section.*
☒	☐	A description of all covariates tested
☐	☒	A description of any assumptions or corrections, such as tests of normality and adjustment for multiple comparisons
☐	☒	A full description of the statistical parameters including central tendency (e.g. means) or other basic estimates (e.g. regression coefficient) AND variation (e.g. standard deviation) or associated estimates of uncertainty (e.g. confidence intervals)
☐	☒	For null hypothesis testing, the test statistic (e.g. *F*, *t*, *r*) with confidence intervals, effect sizes, degrees of freedom and *P* value noted<br>*Give P values as exact values whenever suitable.*
☒	☐	For Bayesian analysis, information on the choice of priors and Markov chain Monte Carlo settings
☒	☐	For hierarchical and complex designs, identification of the appropriate level for tests and full reporting of outcomes
☒	☐	Estimates of effect sizes (e.g. Cohen's *d*, Pearson's *r*), indicating how they were calculated

Our web collection on statistics for biologists contains articles on many of the points above.

Software and code

Policy information about availability of computer code

Data collection	All data collected with analytical instruments mentioned in the methods used the manufacturer's recommended software. The grain analyzer captured individual kernel weights using a custom Visual Basic 6.0 program as described in Spielbauer et al (2009) Cereal Chem. 86(5):556–564.
Data analysis	Data were analyzed with the following published software: Cutadapt v1.1, Trimmomatic v0.22, GSNAP Version 2013-07-20, DESeq2 Bioconductor package, agriGO v2.0, bowtie2 v2.2.853, samtools, GEM v2.5, Integrative Genomics Viewer, meme-chip v4.12.0, bedtools v2.24.0, MotifStack60, Morpheus, ClustalW, blast, Primer3 v. 0.4.0, SAS, R studio, and Excel.

For manuscripts utilizing custom algorithms or software that are central to the research but not yet described in published literature, software must be made available to editors and reviewers. We strongly encourage code deposition in a community repository (e.g. GitHub). See the Nature Portfolio guidelines for submitting code & software for further information.

Data

Policy information about availability of data

All manuscripts must include a data availability statement. This statement should provide the following information, where applicable:

- Accession codes, unique identifiers, or web links for publicly available datasets
- A description of any restrictions on data availability
- For clinical datasets or third party data, please ensure that the statement adheres to our policy

RNA-seq and DAP-seq data are available through the NCBI Accession GSE183304. The ded1-ref mutant is available at the Maize Genetics Cooperative Stock Center.

Field-specific reporting

Please select the one below that is the best fit for your research. If you are not sure, read the appropriate sections before making your selection.

☒ Life sciences ☐ Behavioural & social sciences ☐ Ecological, evolutionary & environmental sciences

For a reference copy of the document with all sections, see nature.com/documents/nr-reporting-summary-flat.pdf

Life sciences study design

All studies must disclose on these points even when the disclosure is negative.

Sample size	Samples sizes for each assay were based on minimum statistical power required to observe mean or frequency differences between homozygous mutant, heterozygous mutant, homozygous normal genotypes or between mutant and normal phenotypes. Sample sizes varied based on the statistical hypothesis being tested. A biological replicate was considered an individual plant and corresponding ear. Individual kernels from single ears were pooled when additional tissue was needed for specific assays. Specific pooling and sampling strategies are described for each experiment presented in the paper.
Data exclusions	Genomics data were filtered for quality and sufficient read coverage as described in the methods. Analytical methods such as PCR markers, EMSA, histology, etc. included quality controls for methodology. Data from analyses that failed to meet quality control metrics (e.g. known genotypes failed to amplify or did not show polymorphism for a primer pair) were excluded from the study.
Replication	The inheritance patterns described were tested with multiple alleles in two inbred genetic backgrounds. Conclusions from genomics data sets were validated with independent experimental approaches such as qRT-PCR from additional biological samples or EMSA.
Randomization	Samples were randomized via genetic segregation (on ears or through random selection of sibling kernels) as well as natural variation in flowering time among sibling plants.
Blinding	Blinding and randomization was incorporated into experiment design when relevant and is described in the methods. Primary analyses of RNA-seq and DAP-seq were completed by independent investigators. Integration and validation of genomics analyses was completed by a third lab group.

Reporting for specific materials, systems and methods

We require information from authors about some types of materials, experimental systems and methods used in many studies. Here, indicate whether each material, system or method listed is relevant to your study. If you are not sure if a list item applies to your research, read the appropriate section before selecting a response.

Materials & experimental systems

n/a	Involved in the study	
☒	☐	Antibodies
☒	☐	Eukaryotic cell lines
☒	☐	Palaeontology and archaeology
☐	☒	Animals and other organisms
☒	☐	Human research participants
☒	☐	Clinical data
☒	☐	Dual use research of concern

Methods

n/a	Involved in the study	
☐	☒	ChIP-seq
☒	☐	Flow cytometry
☒	☐	MRI-based neuroimaging

Animals and other organisms

Policy information about studies involving animals; ARRIVE guidelines recommended for reporting animal research

Laboratory animals	The study did not involve laboratory animals.
Wild animals	The study did not involve wild animals.
Field-collected samples	Field grown maize was planted at the University of Florida Plant Science Research and Education Unit in Citra, FL. Field seasons were grown from mid-March to mid-July and late August to mid-January with supplemental overhead irrigation and conventional agronomic management for fertilizer, herbicides, and pesticides. Exact conditions varied based on plot and environmental conditions for the specific field seasons. Weather data for the Citra, FL field site are available at: https://fawn.ifas.ufl.edu/. Ears from controlled pollinations were harvested by hand, labeled, and dried at 37 °C for 7-10 days. Ears were stored at 10 °C at 50% relative humidity.
Ethics oversight	Interstate movement and field growth of transgenic plants was regulated by the USDA Animal and Plant Health Inspection Service. Recombinant DNA and microbiology experiments were regulated by the Biological Safety Office in the Division of Environmental Health and Safety at the University of Florida.

Note that full information on the approval of the study protocol must also be provided in the manuscript.

ChIP-seq

Data deposition

☒ Confirm that both raw and final processed data have been deposited in a public database such as GEO.

☒ Confirm that you have deposited or provided access to graph files (e.g. BED files) for the called peaks.

Data access links<br>*May remain private before publication.*	https://www.ncbi.nlm.nih.gov/geo/query/acc.cgi?acc=GSE183304
Files in database submission	GSE183302_Ded1_RNAseq_processed_data_file.xlsx; GSE183304_RAW.tar (Narrowpeak and BigWig files); GSE183303_Ded1_DAPseq_processed_data_file.xlsx
Genome browser session<br>(e.g. UCSC)	https://genome.ucsc.edu/s/amarksettles/zm3

Methodology

Replicates	One technical replicate was performed for the HALO-MYB73 DAP-seq experiment.
Sequencing depth	A total of 8,850,971 75bp single-end reads were obtained. Of these, 4,521,987 were uniquely mapped.
Antibodies	DAP-seq used a HALO tagged in vitro transcribed/translated protein. The HALO tag binds a chloroalkane ligand on magentic beads. See https://www.ncbi.nlm.nih.gov/pmc/articles/PMC5576341/ for a detailed methods description of DAP-seq.
Peak calling parameters	DAP-seq reads were trimmed64 and mapped to the B73_v3 reference genome using bowtie2 v2.2.853 (Langmead and Salzberg 2012) with default parameters. Uniquely-mapped reads were kept for further analysis. Peaks were called using GEM v2.5 (Guo et al., 2012) using a Benjamini-Hochberg adjusted p-value (q-value) threshold of q = 0.00001 (option --q 5), while excluding a list of common false positive sites from Galli et al. (2018) Additional background subtraction used sites identified in the HALO-GST in vitro expressed protein sample.
Data quality	Only peaks with an adjusted p-value (FDR) less than 0.00001 (option —-q 5) were retained. Background subtraction was performed using a GST-HALO negative control and blocklist as described in Galli et al., 2018. A total of 43,225 peaks were retained in the final dataset. Of these, all were below a 5% FDR and only three peaks (0.007%) showed less than a 5-fold enrichment.
Software	Reads were aligned using bowtie2v2.3.3. Peaks were called with GEMv2.5. Putative target genes were assigned using Chipseeker v1.10.3. Peaks were visualized using IGV (Robinson et al., 2011). Motif enrichment analysis was performed using GEM and motif logos were produced using MotifStack (Ou et al., 2018).
